# A Review: Preparation, Performance, and Applications of Silicon Oxynitride Film

**DOI:** 10.3390/mi10080552

**Published:** 2019-08-20

**Authors:** Yue Shi, Liang He, Fangcao Guang, Luhai Li, Zhiqing Xin, Ruping Liu

**Affiliations:** 1 School of Printing and Packaging Engineering, Beijing Institute of Graphic Communication, Beijing 102600, China; 2 State Key Laboratory of Advanced Technology for Materials Synthesis and Processing, Wuhan University of Technology, Wuhan 430070, China

**Keywords:** silicon oxynitride, thin film, photoluminescence, chemical vapor deposition, physical vapor deposition

## Abstract

Silicon oxynitride (SiN*_x_*O*_y_*) is a highly promising functional material for its luminescence performance and tunable refractive index, which has wide applications in optical devices, non-volatile memory, barrier layer, and scratch-resistant coatings. This review presents recent developments, and discusses the preparation methods, performance, and applications of SiN*_x_*O*_y_* film. In particular, the preparation of SiN*_x_*O*_y_* film by chemical vapor deposition, physical vapor deposition, and oxynitridation is elaborated in details.

## 1. Introduction

Silicon oxynitride (SiN*_x_*O*_y_*) is an important inorganic material widely studied for its outstanding electronic and mechanical performance. SiN*_x_*O*_y_* is the intermediate phase between silicon dioxide (SiO_2_) and silicon nitride (Si_3_N_4_) [1,2], which possesses high durability at high temperature, high resistance to thermal-shock and oxidation, high density, excellent mechanical performance, and a low dielectric constant [3,4,5,6,7,8]. Due to these unique performance, SiN*_x_*O*_y_* has great potential for high-temperature related applications, for example, it is typically used in non-linear optics [9] and as mechanical component owing to its high strength, thermal insulation, and electronic and chemical resistance [10,11]. When Si_3_N_4_ thin film is utilized as the core material of waveguide devices, Si_3_N_4_ waveguides have better tolerance to sidewall roughness and geometric variations than silicon waveguides, and its relative refractive index difference can increase to 0.62, allowing for much tighter bending radius. Tighter bending radius will not only reduce the bending radius and improve the device integration, but also narrow the waveguide and reduce the input loss [12,13,14,15,16]. However, Si_3_N_4_ has low fracture toughness and poor electrical insulation, limiting its wide applications. SiO_2_ film has a wide range of applications, from microelectronics [17,18] to optical waveguides [19], due to its low dielectric constant, low defect density, and low residual stress. However, SiO_2_ does not perform as an encapsulation layer since oxygen, sodium, and boron can diffuse within it [20,21], which is different from Si_3_N_4_. For these mentioned applications, SiN*_x_*O*_y_* is a more promising candidate, in addition, it has tunable optical and electrical performance. By varying the chemical composition of SiN*_x_*O*_y_*, during the fabrication process, its refractive index and dielectric constant will be tuned [22,23,24]. Except the tunable refractive index, SiN*_x_*O*_y_* also has adjustable thin film stress [25] and exhibits photoluminescence (PL) in the visible light range at room temperature [26]. Thus, SiN*_x_*O*_y_* is highly attractive in integrated circuits (IC) [27], barrier layers [28,29], non-volatile memory [30], optical waveguides [31], organic light emitting diode (OLED) [32], and anti-scratch coatings [33,34]. This review presents a discussion and summary of the preparation methods, performance, and applications of SiN*_x_*O*_y_* film.

## 2. Performance of SiN*_x_*O*_y_* Film

### 2.1. Luminescent Performance

With the development of semiconductor technology, silicon-based micro/nano devices with applications in optoelectronics and IC are in the rapid development. Optoelectronic integration technology urgently requires high-efficiency and high-intensity luminous materials, and the currently-existing silicon integration technology is utilized to develop high-performance optoelectronic devices/systems. Previously, the research on PL of porous silicon and nano-scale silicon at room temperature has aroused widespread attention in this field [34,35,36]. However, porous silicon exhibits several disadvantages such as degradation and poor stability, and it is the most important that it isvery difficult to use it on standard CMOS circuits, thin film sensors, or flexible substrates [37,38]. In addition, nano-scale silicon has problems such as insufficient density, difficulty in controlling size and distribution, unbalanced carrier injection efficiency, complicated luminescence mechanism, and non-radiative recombination [39,40,41,42,43]. As known to all, SiN*_x_*O*_y_* film is an important protective and barrier film with its luminescence characteristics, high mechanical performance, and high reliability [44,45]. Therefore, it is of great significance to study the luminescence characteristics of SiN*_x_*O*_y_* film. Some reported results showed that the luminescence mechanism of SiN*_x_*O*_y_* film is generally divided into three types: defect-state radiation composite luminescence [46], band-tail (BT) radiation composite luminescence [47] and quantum dot radiation composite luminescence [48].

**Figure 1:**
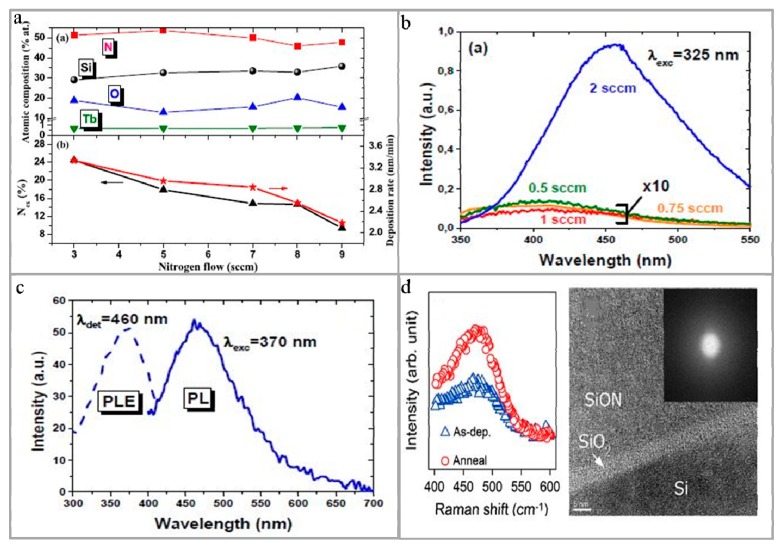
The luminescent performance of SiN*_x_*O*_y_* film. (**a**) Above part shows the effect of flow rate of N_2_ on the atomic composition of the Tb-doped nitrogen-rich SiN*_x_*O*_y_* film, and below part shows the effect of the flow rate of N_2_ on the nitrogen excess parameter (Nex) and deposition rate of the deposited layer [53]. Copyright 2017, *Nanotechnology*. (**b**) Effect of flow rate of N_2_ on the PL of the Ce-doped SiN*_x_*O*_y_* film. (**c**) PL spectrum (solid line) and photoluminescence excitation spectrum (PLE) (dashed line) of a Ce-doped SiN*_x_*O*_y_* film at flow rate of N_2_ of 2 sccm [55]. Copyright 2018, *Nanoscale*. (**d**) Effect of annealing temperature on SiN*_x_*O*_y_* film [46]. Copyright 2013, *J. Lumin*.

The light-emitting performance of SiN*_x_*O*_y_* film is effected by its composition, because its composition has significant influences on the formation of Si-O, Si-N, Si-H, and N-H bonds, resulting in the changes of absorption peak position of SiN*_x_*O*_y_* film [49,50]. As a result, many researchers studied the atom ratio of N and O in SiN*_x_*O*_y_* film [51,52]. During the preparation of SiN*_x_*O*_y_* film, it is found that the luminescence performance of the SiN*_x_*O*_y_* film can be adjusted by changing the flow rate of nitrogen (N_2_) and concentration of oxygen. In order to investigate the effect of flow rate of N_2_ on the evaporated SiN*_x_*O*_y_* film, Lee et al. [53] prepared a SiN*_x_*O*_y_* film on a poly(ethylene naphthalate) (PEN) substrate by ion-beam assisted electron beam evaporation at room temperature. It is found that when the flow rate of N_2_ is 40 sccm, the refractive index of SiN*_x_*O*_y_* film increased to 1.535, and the SiN*_x_*O*_y_* film density increased to ~2.5 g cm^−3^, whereas the surface roughness and optical transmittance of SiN*_x_*O*_y_* film decreased. In preparation of the SiN*_x_*O*_y_* film, adjusting the flow rate of N_2_ has a significant improvement on the luminescent performance of the doped SiN*_x_*O*_y_* film. Among them, Labbé et al. [54] prepared a nitrogen-rich SiN*_x_*O*_y_* film doped with Tb^3+^ by reactive magnetron co-sputtering under N_2_ with different flow rates and annealing conditions. The influences of flow rate of N_2_ on the atomic composition of SiN*_x_*O*_y_* film, the N excess (N_ex_) in the sedimentary layer and the deposition rate are shown in Figure 1a. For the synthesis, the reverse flow of N_2_ during deposition is studied. Through the characterization results, the researchers carefully identified different vibration modes of Si-N and Si-O bonds, especially the ‘non-phase’ tensile vibration mode of Si-O bonds. The highest PL intensity of Tb^3+^ is obtained by optimizing the nitrogen incorporation and annealing condition. The aggregation effect in Si_3_N_4_ matrix is significantly reduced, thus allowing the higher concentration of optically active Tb^3+^, which promoted its luminescence applications. In related researches, Ehré and co-workers [55] prepared a cerium (Ce)-doped SiN*_x_*O*_y_* film by magnetron sputtering under N_2_ atmosphere. Their results showed that a broad and strong PL peak is red-shifted at the N_2_ flow rate of 2 sccm. The peak of PL band shifts to 450 nm, and the results showed that the PL strength is 90 times higher than the BT strength of the sample deposited at a low flow rate of N_2_. The effects of flow rate of N_2_ on PL of Ce-doped SiN*_x_*O*_y_* film, PL spectrum (solid line), and PL excitation spectrum (dotted line) at flow rate of N_2_ (2 sccm) are shown in Figure 1b,c. As described above, it is possible to adjust not only the luminescence characteristics of the SiN*_x_*O*_y_* film by changing the flow rate of N_2_ but also the oxygen content, such as Huang et al. [56] demonstrated the strong PL of SiN*_x_*O*_y_* film by adjusting the oxygen content. With the oxygen content in the SiN*_x_*O*_y_* film increasing from 8% to 61%, the PL changed from red light to orange light and white light, and they indicated that the change in PL performance of SiN*_x_*O*_y_* film is due to the change in the center of the defect luminescence and the change in the main phase structure from Si_3_N_4_ to SiN*_x_*O*_y_* and SiO_2_. Similarly, the researchers studied the effect of oxygen concentration on PL of SiN*_x_*O*_y_* film doped with other components. Steveler et al. [57] prepared an Er^3+^-doped amorphous SiN*_x_*O*_y_* film by reactive evaporation. They found that the PL of Er^3+^ is observed only in the samples with oxygen concentration equal to or less than 25%, and they indicated that oxygen will make Er^3+^ ions optically active and can be indirectly excited in the presence of excess Si. It is further confirmed that, when both the amounts of oxygen and nitrogen are equal to or about 25%, Er^3+^ related PL increases with the increase of annealing temperature.

Processing conditions of the preparation of SiN*_x_*O*_y_* film also have influences on the PL performance of SiN*_x_*O*_y_* film, such as the annealing process. For instance, the SiN*_x_*O*_y_* film prepared by reactive sputtering on a silicon substrate, and then vacuum-annealed at 900 °C for 1 hour, is amorphous, composed of mixed Si-N and Si-O bonds, and blue and green emission are observed in the PL spectra of this prepared SiN*_x_*O*_y_* film, as shown in Figure 1d [46], so the SiN*_x_*O*_y_* film integrated with a top electrode is used for a electroluminescent device. 

### 2.2. Adjustable Refractive Index

Adjustable refractive index means that the refractive index can be continuously tuned along the normal direction of the surface of the SiN*_x_*O*_y_* film by changing the proportion of the reaction gas [58]. In order to prepare graded-index SiN*_x_*O*_y_* film for applications in optical waveguide materials, gradient-index films, and anti-reflection films, researchers conducted extensive research on the refractive index-adjustable performance of SiN*_x_*O*_y_* film, such as the research on controlling the flow rate and ratio of reactive gas, reaction process, etc. [59,60,61]. Hänninen et al. [62] found that the refractive index and extinction coefficient of the SiN*_x_*O*_y_* film decrease with the increase of oxygen and nitrogen contents in the SiN*_x_*O*_y_* film, which could be adjusted by controlling the ratio of reactive gas. Therefore, in order to control the ratio, they applied reactive high-power pulsed magnetron sputtering to synthesize a SiN*_x_*O*_y_* film using N_2_O as a single source, providing oxygen and nitrogen for SiN*_x_*O*_y_* film’s growth. The characterization results showed that the synthesized SiN*_x_*O*_y_* film has the characteristics of silicon-rich, amorphous, and randomly-chemical-bonding structure. Furthermore, Himmler et al. [32] used reactive magnetron sputtering to deposit SiN*_x_*O*_y_* film and found that the refractive index of SiN*_x_*O*_y_* film depends on its oxygen and nitrogen contents, so they adjusted the refractive index by controlling the content ratio of oxygen/nitrogen. It is found that the reaction gases are differently incorporated into the layer due to different plasma conditions in the coating region, so there is a higher nitrogen incorporation and a higher refractive index in plasma regions with a high plasma density, while plasma regions with lower plasma density will result in a higher oxygen bonding and a lower refractive index. In addition to controlling the proportion of gas, the preparation method also has a certain influence on the refractive index of the SiN*_x_*O*_y_* film. Farhaoui et al. [63] used the reactive gas pulse in sputtering process (RGPP) to adjust the composition of SiN*_x_*O*_y_* film from oxide to nitride by controlling the average flow rate of O_2_. Compared with the conventional reaction process (CP), not only did the deposition rate increase, but also a wide range of SiN*_x_*O*_y_* films’ refractive indexes varying within the same range could be obtained through this pulse process. Moreover, extinction coefficient of the SiN*_x_*O*_y_* film is low, and this SiN*_x_*O*_y_* film can be used for multi-layer anti-reflection coating (ARC). Nakanishi et al. [64] also introduced argon (Ar) in the preparation of SiN*_x_*O*_y_* film by pulsed direct current (DC) reactive magnetron sputtering. They found that the higher the Ar concentration is, the more stable the SiN*_x_*O*_y_* film’s formation and the higher the deposition rate is. The researchers claimed that a large amount of sputtered silicon atoms reach the substrate at high Ar concentration, causing the oxidation probability of the SiN*_x_*O*_y_* film to decrease and the refractive index of the SiN*_x_*O*_y_* film to gradually change with the percentage of oxygen in the reactive gas. The tunable refractive index of SiN*_x_*O*_y_* film makes it superior to Si_3_N_4_ and SiO_2_ in optical device applications. Furthermore, it also provides a new strategy for development of optical devices.

## 3. Preparation of SiN*_x_*O*_y_* Film

At present, the preparation methods of SiN*_x_*O*_y_* film are mainly classified into chemical vapor deposition (CVD), physical vapor deposition (PVD), high-temperature nitridation, and ion implantation [65]. The descriptions and comparisons of these preparation processes are as follows. 

### 3.1. CVD Method

CVD is a vapor phase growth method for preparing materials by introducing one or more compounds containing a constituent film element. During this growth process, the reactive gas is purged into a reaction chamber in which a substrate is placed, and a depositing process on the gas–phase or gas–solid interface is executed to generate solid sediments [66]. CVD based methods are mainly divided into plasma enhanced CVD (PECVD), low-pressure CVD (LPCVD), photochemical vapor deposition [67], thermal CVD [68], etc. Among them, PECVD and LPCVD are the most commonly employed methods. Additionally, PECVD can be extended to radio frequency PECVD (RF-PECVD) [69], electron cyclotron resonance PECVD (ECR-PECVD) [70,71], and inductively coupled PECVD (IC-PECVD) [72]. 

#### 3.1.1. PECVD

PECVD is a method for preparing a semiconductor thin film which is subjected to chemical reaction deposition on a substrate using a glow discharge in a deposition chamber [73]. The preparation process of SiN*_x_*O*_y_* film via PECVD is generally described as follows: at low temperature (<400 °C), ammonia (NH_3_), pure silane (usually SiH_4_), N_2_, and nitrous oxide (N_2_O) are generally employed in a PECVD chamber with a certain power. There are generally some differences in the composition of the precursor gases reported in studies. In general, the flow rates of NH_3_, pure silane, and N_2_ remain the same, and the total flow rate is controlled by adjusting the flow rate of N_2_O [74]. Generally, SiN*_x_*O*_y_* film is deposited on silicon substrate or quartz substrate, wherein the substrate’s temperature is kept at room temperature, but some composite films are deposited on other composite layers by PECVD, such as the preparation of SiN*_x_*O*_y_* and Si_3_N_4_ by Park et al. [75]. For composite film, they deposited a SiN*_x_*O*_y_* film directly on the deposited Si_3_N_4_ layer. 

For SiN*_x_*O*_y_* film by PECVD, NH_3_ is often used as the reaction gas of the nitrogen source, and SiH_4_ is used as the reaction gas of the silicon source. Although NH_3_ reacts with SiH_4_ easily, the SiN*_x_*O*_y_* film produced by NH_3_ at a lower temperature has a higher hydrogen content, which causes the decreased electrical performance of SiN*_x_*O*_y_* film, so some studies used RF-PECVD, with N_2_ and SiH_4_ as precursor gases to prepare SiN*_x_*O*_y_* film with lower hydrogen content, and some studies used RF-PECVD, with N_2_, SiH_4_ and NH_3_ as the front gases [76]. For example, Kijaszek et al. [77] used RF-PECVD and maintained the RF of 13.56 MHz, pressure, power, and substrate temperature (350 °C), and controlled the composition of SiN*_x_*O*_y_* film by the flow ratio of different gaseous precursors: NH_3_, 2% SiH_4_/98% N_2_ and N_2_O, wherein the flow rate of 2% SiH_4_/98% N_2_ and N_2_O remained the same, and the flow rate of NH_3_ is adjusted to control the SiN*_x_*O*_y_* film’s performance. When the flow rate of NH_3_ is low, the SiN*_x_*O*_y_* film’s hydrogen content is also lowered, and the electrical performance of the SiN*_x_*O*_y_* film is improved. 

ECR-PECVD is also included in the PECVD method. Okazaki et al. [78] deposited a SiN*_x_*O*_y_* film under hydrogen-free conditions by ECR-PECVD at a low temperature of ~200 °C with O_2_ and N_2_ as reaction gases, and the obtained deposition rate is ~0.1 μm min^−1^. The deposited SiN*_x_*O*_y_* film has good optical performance. Furthermore, Wood [79] used an ECR-PECVD system to deposit SiN*_x_*O*_y_* dielectric film at low substrate temperature. The electrical performance of these films is found to be comparable with those deposited in systems using ion-assisted PVD and sputtering systems. Furthermore, thin film electroluminescence devices containing ECR SiN*_x_*O*_y_* dielectrics exhibit high brightness and excellent breakdown characteristics.

#### 3.1.2. LPCVD 

For LPCVD, a gas source under low pressure is decomposed to deposit SiN*_x_*O*_y_* film directly on a substrate. Since the mean free path of the reactive gas molecules increases at a low pressure, the diffusion coefficient increases. Thereby, the transmission speeds of gaseous reactants and by-products are increased, the aggregation of impurities on the substrate is reduced to some extent, and the film is more uniform. It has the advantages of structural integrity, few pinhole defects, and high deposition rate, therefore it is suitable for large-area production [80]. Kaghouche et al. [81] deposited a SiN*_x_*O*_y_* film on a single crystal silicon wafer using LPCVD at a high temperature of 850 °C with N_2_O, NH_3_ and dichlorosilane (SiH_2_Cl_2_) as precursor gases. In the synthesis, the control variable experiment is carried out by adjusting the flow ratio of NH_3_/N_2_O via keeping the flow rate of SiH_2_Cl_2_ as constant. Additionally, the deposition duration remains constant to maintain similar annealing conditions during the deposition process. Finally, the thickness of the obtained SiN*_x_*O*_y_* film is generally in the range of 300–400 nm.

However, the LPCVD has its disadvantages of low heating rate, long reaction time, and high deposition temperature (generally >550 °C), which limit its applications to some extent. A comparison of different CVD methods is summarized in Table 1 [76,82,83,84,85]. 

#### 3.1.3. High Temperature Thermochemical Vapor Deposition (HTCVD)

The HTCVD method uses a direct heating method to decompose or chemically react to obtain a solid film on the surface of a substrate [86]. Since the HTCVD method uses direct heating to provide activation energy for the gas, it does not require complicated equipment, and the operation is relatively facile. In addition, the reaction rate is high, and the formed film has less impurity of hydrogen and a dense structure [80]. However, the heating temperature of the HTCVD method is generally high (≥700 °C), which easily causes deformation and internal structural change of the substrate, reducing the mechanical performance of the substrate and the bonding force between film and substrate. 

For optimization of this method, a rapid thermal CVD (RTCVD) is investigated [68]. RTCVD refers to the formation of a single-layer SiN*_x_*O*_y_* film by a rapid annealing method to seal the substrate, thereby avoiding the substrate being affected by high temperature [76]. The RTCVD method can deposit advanced dielectric films on III-V substrates under high temperature. This method not only has the advantages of HTCVD, but also prevents the V group elements from sublimating due to high temperature. Lebland et al. [87] deposited SiN*_x_*O*_y_* film on III-V substrates by RFCVD. They controlled the deposition rate and stoichiometry of the SiN*_x_*O*_y_* film by adjusting the partial pressure of N_2_O and temperature. It is found that a deposition rate of up to 10 nm s^−1^ is obtained at 750 °C, and the InP substrate does not degrade, solving the contradiction between the high deposition temperature using the direct CVD method and the degradation of the V group element.

#### 3.1.4. Photochemical Vapor Deposition (Photo-CVD)

Photo-CVD is a novel low-temperature deposition method using ultraviolet (UV) light or laser to photodecompose a reaction gas to obtain a solid film [88]. It has the following advantages: greatly reducing the substrate temperature (≤250 °C), avoiding the damage caused by high-energy particle radiation on the surface of the film, making the surface of the film smooth, and reducing the by-product [89]. However, the main disadvantages of the photo-CVD are its high cost, and that the formed film is not stable.

The CVD method has certain advantages and many extension methods, Table 2 shows the pros and cons of four different CVD approaches [80,89,90,91,92], among them PECVD and LPCVD are the most commonly used deposition methods. Even so, it is difficult to avoid the existence of hydrogen in preparing SiN*_x_*O*_y_* film by PECVD or LPCVD, and the hydrogen deposited in the raw material is difficult to remove due to the low deposition temperature of the CVD method, further affecting the performance of SiN*_x_*O*_y_* film [90]. To this end, researchers have proposed various methods to reduce the hydrogen content of SiN*_x_*O*_y_* film. Among them, the thermal oxidation treatment by Hallam et al. [91] is highly promising, they deposited a SiN*_x_*O*_y_* film by PECVD and demonstrated that the SiN*_x_*O*_y_* film with Si-H peak wave number of >2200 cm^−1^ has an open circuit voltage of up to 80 mV during thermal annealing. They pointed out that the result is due to an increase in oxygen content. In addition, increasing the flow of raw materials and the annealing temperature will also reduce the hydrogen content to various degrees.

### 3.2. PVD

PVD is a physical method of vaporizing a material source into gaseous atoms, molecules, or ionized ions under vacuum conditions, and depositing a film on the substrate by sputtering or plasma technology [93]. The main processes of PVD are vacuum evaporation, sputter coating, ion plating, arc plasma coating, etc. [94,95,96]. Among them, sputter coating is a widely used and mature method, which means that under vacuum conditions, the surface of the target material is bombarded with the particles having the function, then the surface atoms of the target are obtained with sufficient energy to escape, and the sputtered target is deposited on the substrate to form a film [97]. The large-scale magnetron sputtered coating developed on the basis of sputter coating has high deposition rate, good process repeatability, easy automation, etc. [90].

**Figure 2:**
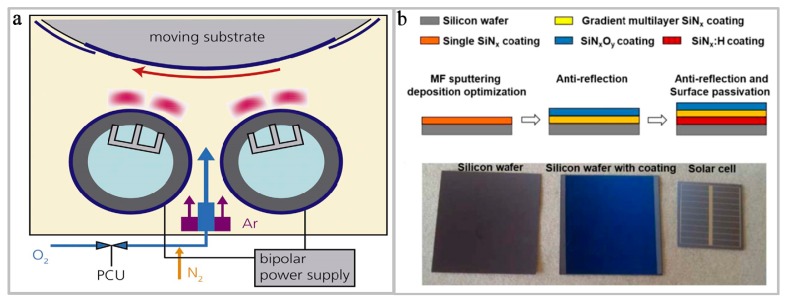
Physical vapor deposition (PVD). (**a**) Schematic of the deposition zone inside the roll-to-roll tool [32]. Copyright 2018, *Surf. Coat. Technol*. (**b**) The procedure design and application for laboratory silicon solar cell of the anti-reflection and surface passivation film [101]. Copyright 2017, *Mater. Sci. Semicond. Process*.

Magnetron sputtering coating method is also divided into several categories such as reactive pulsed magnetron sputtering, pulsed DC magnetron sputtering, rotatable dual magnetron pulsed DC reactive magnetron sputtering, intermediate frequency (MF) magnetron sputtering, etc. [98]. Tang et al. [99] deposited SiN*_x_*O*_y_* film by reactive pulsed magnetron sputtering, and the effects of nitrogen ratio on the optical, structural, and mechanical performance of SiN_x_O_y_ film are investigated. They found that with the increase of nitrogen ratio, the refractive index of the SiN*_x_*O*_y_* film increased from 1.487 to 1.956, its surface roughness decreased from 1.33 to 0.97 nm, its hardness increased from 13.51 to 19.74 GPa, and its Young’s modulus increased from 110.41 to 140.49 GPa. When the SiN*_x_*O*_y_* film is applied to the anti-reflection coating, the hardness of the coating is greatly improved. Additionally, Simurka et al. [100] deposited a SiN*_x_*O*_y_* film on a glass substrate by pulsed DC magnetron sputtering with a constant flow of Ar (38–94 sccm) as the working gas and a constant gas flow ratio of oxygen (2–5 sccm) and nitrogen (20 sccm) is employed. Through experimental characterizations, it is found that as the sputtering power increases, the density, refractive index, hardness, and Young’s modulus of the film increase slightly. Therefore, in the preparation process, it is very important to select the appropriate sputtering power according to the requirements. Furthermore, in the preparing process of SiN*_x_*O*_y_* film, not only a reasonable sputtering power should be set, but also the voltage is important. Himmler et al. [32] deposited a single layer of SiO*_x_*, SiN*_y_*, and SiN*_x_*O*_y_* using a rotatable dual magnetron pulsed DC reactive magnetron sputtering, as shown in Figure 2a, showing a layout of deposition zone within the roll-to-roll coating tool. Experimental results demonstrated that the ratio of oxygen/nitrogen in the layer is determined not only by the reactive gas, but also by the voltage. Furthermore, Li et al. [101] deposited a multilayer film of hydrogenated silicon nitride (SiN*_x_*:H)/silicon nitride (SiN*_x_*)/SiN*_x_*O*_y_* for silicon crystal solar cells using intermediate frequency (MF) magnetron sputtering, and the process design and application of passivation film on anti-reflection surface of silicon solar cell in laboratory are shown in Figure 2b. The multilayer film prepared by this process has excellent cell efficiency when applied to solar cells.

Although the SiN*_x_*O*_y_* film prepared by the PVD method has a lower hydrogen content than that of the SiN*_x_*O*_y_* film by CVD, the PVD is superior to the CVD for preparing SiN*_x_*O*_y_* film. Moreover, the sputtering method has a low deposition temperature and is easy to control. However, it is difficult to perform rapid film deposition on a large-scale substrate by sputtering method [102] and the sputtering method is prone to “target poisoning” [103], that is the added reaction gas generates a composite material on the target, thereby changing the sputtering yield and the deposition rate. Finally, the film stoichiometry is changed. 

### 3.3. Oxynitridation

Oxynitridation is generally a method of reacting gas such as N_2_O, NO, NH_3_, N_2_, and O_2_ with Si, SiO_2_, or Si_3_N_4_ under suitable conditions to obtain a SiN*_x_*O*_y_* film.

The high-temperature nitridation is a more commonly used method, which refers to the preparation of SiN*_x_*O*_y_* film by introducing N into SiO_2_ film by means of thermal nitriding [104], rapid annealing [105], and plasma nitriding [106] under certain conditions with reactive gas containing nitrogen (e.g., N_2_O, NO, NH_3_, N_2_) [107]. Among them, a SiO_2_ film which can be obtained by a thermal oxidation method [108] and a sol–gel method [109]. Different performance of membranes prepared under different oxidation/nitridation conditions in the nitrogen oxidation process are obtained. When the temperature is 800–1200 °C, NH_3_ is introduced as a nitrogen source for high-temperature nitridation of SiO_2_ by thermal nitridation, although a simple and smooth SiN*_x_*O*_y_* film can be obtained, H is inevitably introduced. H doping in the SiN*_x_*O*_y_* film affects its electrical performance, such as the formation of a large number of electron traps in the SiN*_x_*O*_y_* film. In order to reduce the effect of H on membrane performance, reoxidation is an effective method [110]. Furthermore, a nitrogen-containing gas without H can be used as a nitrogen source, such as N_2_O, to form a Si-O-N film in situ generation without introducing a H atom. However, the high temperature conditions of the high temperature nitridation process have a serious influence on the substrate and are prone to defects. In addition, the SiN*_x_*O*_y_* film obtained by this method has poor uniformity and poor compactness.

Three methods for preparing SiN*_x_*O*_y_* film are introduced above, and the advantages and disadvantages of different preparation methods are summarized in Table 3 [110,111,112,113].

**Figure 3:**
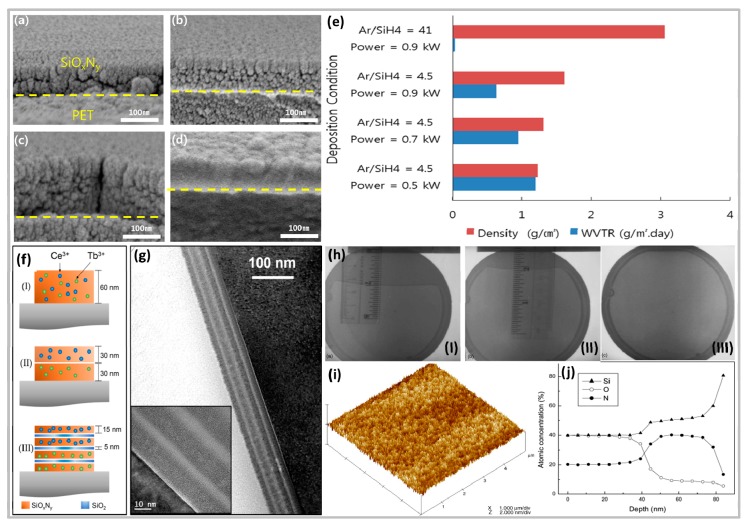
The surface morphology and performance of SiN*_x_*O*_y_* film are changed under different conditions. (**a**–**d**) FESEM cross-section images of SiN*_x_*O*_y_* film deposited at Ar/SiH_4_, O_2_/(O_2_+N_2_) of 4.5 and 0.1, respectively with RF power of (a) 0.5 kW, (b) 0.7 kW, (c) 0.9 kW, and (d) at Ar/SiH_4_, O_2_/(O_2_ + N_2_) of 41 and 0.1, respectively with RF power of 0.9 kW. (**e**) Effect of Ar/SiH_4_ ratio and RF power variation on the density and WVTR of 100 nm-thick SiN*_x_*O*_y_* film deposited at O_2_/(O_2_ + N_2_) ratio of 0.1 [72]. Copyright 2019, *Thin Solid Films*. (**f**) Sample description, being (I) a Ce^3+^ and Tb^3+^ co-doped SiN*_x_*O*_y_* film, 60 nm thick with 42 at.% of N; (II) a bilayer composed of a Ce^3+^ doped SiN*_x_*O*_y_* single layer and a Tb^3+^- doped SiN*_x_*O*_y_* single layer, each 30 nm thick; and (III) a multilayer made of two layers of Tb^3+^- doped SiN*_x_*O*_y_* and two layers of Ce^3+^-doped SiN*_x_*O*_y_* (each sub-layer with a thickness of 15 nm), separated by SiO_2_ spacers of 5 nm. All SiN*_x_*O*_y_* layers have a nitrogen content of 42 at. %. (**g**) HRTEM image of the multilayer design (structure (III), as-deposited sample). The inset shows a magnified region of the multilayer close to the Si substrate, for a sample annealed at 1180 °C for 1 h [114]. Copyright 2016, *J. Appl. Phys*. (**h**) Infrared transmission image of a bonded pair after the crack-opening test: (I) as-bonded, (II) after annealing at 120 °C, (III) after annealing at 300 °C. (**i**) AFM image of SiN*_x_*O*_y_* film synthesized by plasma immersion ion implantation (PIII). (**j**) Depth profiles of Si, O, and N in SiN*_x_*O*_y_* film acquired by sputtering XPS [115]. Copyright 2005, *Appl. Surf. Sci.*

The deposition process of the SiN*_x_*O*_y_* film is reviewed. The properties of the film microstructure for different growth conditions and post-growth thermal annealing will be illustrated by Figure 3. As shown in Figure 3a–c, when the ratio of Ar/SiH_4_ and O_2_/(O_2_ + N_2_) is 4.5 and 0.1, respectively, the porosity of the SiN*_x_*O*_y_* film gradually decreases. However, at this state, the SiN*_x_*O*_y_* film is still porous and pore-oriented. The effect of the rate reduction is small. As shown in Figure 3d, by changing Ar/SiH_4_ from 4.5 to 41 and RF to 0.9 KW, a very significant decrease in porosity is observed, showing a very dense SiN*_x_*O*_y_* film, and it is shown by Figure 3e when Ar/SiH_4_ changes from 4.5 to 41, and when the RF is 0.9 KW, its water vapor transmission rate (WVTR) is the lowest, and it has good protection function [72]. Figure 3f,g shows HRTEM images of different types of Ce^3+^ and Tb^3+^ co-doped SiN*_x_*O*_y_* film and structure (III) multilayer designs. It is worth noting that no ripples and layer mixing are observed. Furthermore, the fact that the morphological structure remained unchanged even after annealing at 1180 °C for 1 h confirmed the manufacturing process and showed the robustness of the sample (Figure 3g) [114]. As shown in Figure 3h, (I) and (II) are infrared transmission photographs of the bond pairs and the annealed pairs, respectively. The results show that the crack length of the composite is obviously shortened after annealing at 120 °C, and the bond strength is obviously improved. After the sample is annealed at 300 °C for 2 h, the bond strength is sufficient to withstand peeling, and the infrared transmission photograph is shown in Figure 3 h(III). As shown in the AFM chart (Figure 3i), the SiN*_x_*O*_y_* film has a smooth and uniform surface with a root mean square (RMS) roughness of 0.162 nm, which is sufficient for direct soldering without complicated grinding and polishing processes. Figure 3j shows the corresponding depth profiles of N, O, and Si. Obviously, you can find two layers. From the surface to a depth of about 40 nm, the amounts of O and N are about 40% and 20%, respectively. Starting from 40 nm, the O concentration gradually decreases compared with the first layer, but the concentration of N increases. After annealing at 1100 °C, the higher structural disorder confirmed by XRD analysis in the SiN*_x_*O*_y__y_* film can be attributed to the presence of O. This helps to inhibit polycrystallization of the buried insulator during post-annealing in conventional CMOS processes [115]. By analyzing the effects of different growth conditions and the properties of the thermally annealed film microstructure by post-growth on the performance of SiN*_x_*O*_y_* film, it is helpful to effectively control the parameters as needed in later applications.

## 4. Applications of SiN*_x_*O*_y_* Film

**Figure 4:**
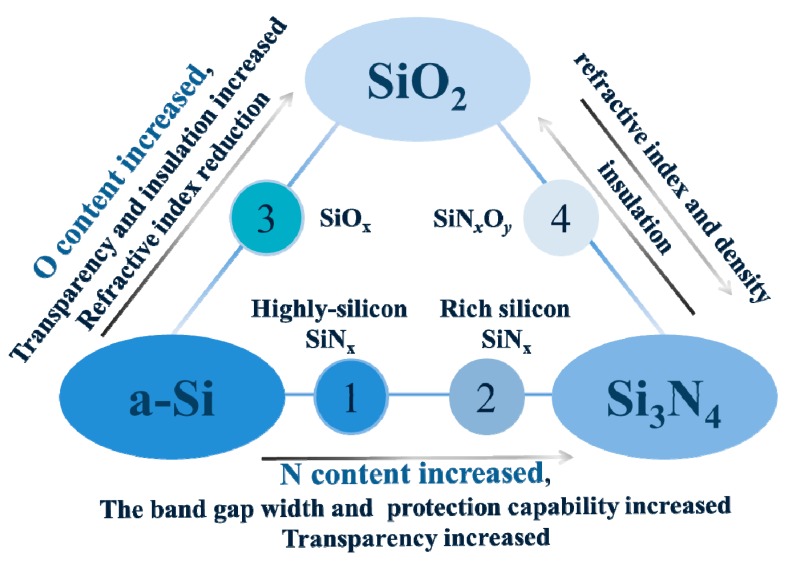
A series of possible forms of the SiN*_x_*O*_y_* film and their performance.

As an intermediate of SiN*_x_* and SiO_2_, SiN*_x_*O*_y_* film has an adjustable dielectric constant, refractive index, and extinction coefficient by controlling the ratio of nitrogen/oxygen in the chemical composition [116]. The gas ratio and process parameters of the various reactants involved in the preparation of SiN*_x_*O*_y_* are different, so that the stoichiometry of each element in SiN*_x_*O*_y_* is different, and the limit forms may be a-Si, SiO_2_, and Si_3_N_4_.As shown in the Figure 4, a series of possible forms of the SiN*_x_*O*_y_* film is given, as well as changes in the properties of different film forms as the O content and H content increase, are mainly reflected in changes in transparency, band gap width, refractive index, and insulation [117]. SiN*_x_*O*_y_* film has performance between SiO_2_ and Si_3_N_4_. Due to its excellent photoelectric performance, it has been widely used in optical devices, dielectric gate dielectric materials, and optical waveguide materials [118]. The SiN*_x_*O*_y_* film also has high chemical stability, high resistance to impurity diffusion, and water vapor permeability, which is highly promising for applications in barrier devices such as gas barriers [119]. In addition, the SiN*_x_*O*_y_* film has a small defect density and is advantageous in applications as a storage medium. The applications of SiN*_x_*O*_y_* film in microelectronic devices, optical devices, barrier materials, and non-volatile memory will be introduced in this section.

### 4.1. Application of Barrier Material

Most organic conductive polymers and chemically reactive electrodes degrade when exposed to water or oxygen, causing failure of electronic devices [120]. Therefore, in order to enhance the service life of device, it is necessary to use a barrier material for encapsulation layer of the device with a low water vapor transmission rate and oxygen permeability. Currently, glass or metal is often used for device packaging, however, these rigid materials with high spring constant will greatly limit the widespread applications of devices, especially in flexible devices. On this basis, researchers studied thin-film encapsulation (TFE) technology [121]. In recent years, the SiN*_x_*O*_y_* film has also been widely used as encapsulating layers. However, SiN*_x_*O*_y_* film has some problems served as an encapsulation layer. The basic degradation mechanism of water vapor or oxygen on a single layer of SiN*_x_*O*_y_* film is studied, and it is found that water vapor diffuses into the SiN*_x_*O*_y_* film through the percolation channel and nano-defects to react with SiON:H, making Si-N gradually changes to Si-O bond under repeated erosion of water vapor, eventually becoming a fully oxidized film. Therefore, the degradation of SiO_2_:H becomes a way for water vapor to diffuse, and because of the lower density of SiO_2_:H, the diffusion rate of water vapor in the SiN*_x_*O*_y_* film is further enhanced [116]. In addition, the difference in moisture resistance of the SiN*_x_*O*_y_* film is explained by film oxidation and surface defect density. Oku and co-workers [122] indicated that the surface of SiN*_x_*O*_y_* film easily changes to a low-quality film containing an excessive amount of H_2_O molecule and O-H bond, which reduces the waterproofness, after pressure cooker test (PCT) test. Therefore, it is generally formed by depositing a multilayer film to reduce film defects and the probability of film pores communicating with the atmosphere, thereby forming a perfect barrier layer. On the other hand, SiN*_x_*O*_y_* film deposited at a higher temperature has higher performance, and its defects are less, however, the high-temperature treatment should be avoided in some devices, and the deposited SiN*_x_*O*_y_* film has poor barrier performance under low temperature, which is not conducive to the protection of the device. Therefore, Satoh et al. [123] developed a AlO*_x_*/SiON composite bilayer structure deposited at a relatively low temperature (<160 °C), in which the AlO*_x_* layer is used as a barrier layer, and the SiN*_x_*O*_y_* layer ensured the chemical and thermal stability of the film to protect the device from damage during processing. The test results indicated that the device did not show any degradation at 85 °C, and 85% relative humidity (RH) is obtained.

Additionally, some researchers have developed other methods, such as changing the preparation process and controlling the preparation parameters, to optimize the barrier performance of SiN*_x_*O*_y_* film to air and water vapor. Shim et al. [124] deposited a thin SiN*_x_*O*_y_* layer on the surface of poly(ether sulfone) (PES) membrane by PECVD using a mixture of hexamethyldisiloxane (HMDSO) and ammonia. SiN*_x_*O*_y_* is used as gas barrier layer, and a silicon-based coating layer of an organic/inorganic hybrid structure is added between the surface of PES and SiN*_x_*O*_y_* film as a buffer layer for both. The results showed that the undercoat layer is indispensable in the composite film. Under the action of the undercoat layer, the dense inorganic SiN*_x_*O*_y_* layer has an excellent oxygen barrier performance of 0.2 cm^3^ m^−2^ day^−1^ at a critical coating thickness of ~20 nm. Moreover, the presence of the undercoat layer not only prevents the film from being cracked when the composite film is highly curved, but also maintains the initial gas barrier performance of the curved film. Iwamori et al. [125] prepared a SiN*_x_*O*_y_*-based transparent gas barrier material by depositing a SiN*_x_*O*_y_* film on a polyethylene terephthalate (PET) substrate by reactive sputtering in a nitrogen plasma. The results showed that the SiN*_x_*O*_y_* film has a lower oxygen transmission rate than that of the SiO_2_ film, because the use of nitrogen plasma instead of oxygen suppresses the formation of defects induced by peroxide, and the increase in nitrogen content greatly enhances the density of SiN*_x_*O*_y_* film, forming a composite film with excellent gas barrier performance. Liu et al. [126] deposited a SiN*_x_*O*_y_* film on PES by RF magnetron sputtering in Ar/N_2_ atmosphere as a barrier to prevent water vapor permeation. The experimental results showed that the RF power is 250 W. At the fixed film pressure of 1.6 Pa, with the deposition time of 30 min, the 100% N_2_ content, and the absence of substrate bias, the water vapor transmission rate of the composite film is two orders of magnitude smaller than that of the uncoated PES.

These studies provided some new research ideas for the development of high-quality SiN*_x_*O*_y_* barrier material, and have important research significance for the applications of SiN*_x_*O*_y_* film in packaging materials, protective layer materials, and gas barriers.

### 4.2. Application of Non-Volatile Semiconducting Memory

Non-volatile semiconducting memory (NVSM) means that when the current is turned off, the stored data does not disappear, which can be applied to many fields [127,128,129]. Wrazien et al. [130] conducted a lot of work on non-volatile memory. They studied NVSM with silicon-oxide-nitride-oxide-silicon (SONOS) structure, which theoretically proves its storage capacity under low pressure and 150 °C can be as long as 10 years and the erase/write cycle is 10^5^ times. Moreover, the SONOS based device has an oxynitride charge storage layer. In recent years, resistive memory is considered as a non-volatile memory with broad application prospects because of its excellent durability, high data transmission speed, and low power consumption [131]. Resistive random access memory (RRAM) is also known as a memristor-an electronic component with memory function [132]. Currently, excellent resistance switching characteristics of silicon-based dielectrics are often applied in RRAM devices. Recently, with the development of SiN*_x_*O*_y_* film, it has been found that the defect density of SiN*_x_*O*_y_* film is low, and the high operating voltage is more advantageous when used as a storage medium. Chen et al. [133] proposed that the SiN*_x_*O*_y_* film has non-uniformities such as operating voltage and current, and improved uniformity of the RRAM by doping. They prepared SiN*_x_*O*_y_* film with different oxygen concentrations by reactive magnetron sputtering. The resistance conversion performance and conduction mechanisms of Cu/SiN_x_O_y_/ITO device are investigated. The SiN*_x_*O*_y_* is deposited under O_2_ with a flow rate of 0.8 sccm in their investigation. The fabricated Cu/SiN*_x_*O*_y_*/ITO device showed reliable resistance conversion behavior, including high durability and retention. Zhang et al. [134] prepared a SiN*_x_*O*_y_* film with ultra-low power a-SiN_0.69_O_0.53_:H by PECVD and pass N_2_O into a vacuum chamber. An a-SiN_0.69_O_0.53_:H film is prepared under high resistance state and low power conversion. The RRAM cells based on the a-SiN_0.69_O_0.53_:H film shows an ultra-low current compared with the pure a-SiN_0.62_:H-based RRAM, effectively reducing the operating current. 

**Figure 5:**
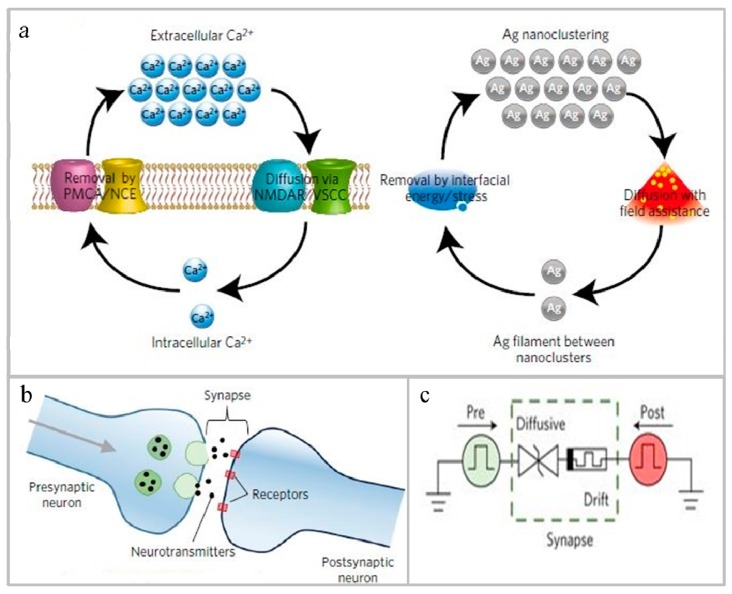
Resistive random-access memory (RRAM) that mimics synaptic memory. (**a**) Schematic illustration of the analogy between Ca^2+^ and Ag dynamics. (**b**) Illustration of biological synaptic connections between presynaptic and postsynaptic neurons and circuit diagrams of electrical synapses [135]. Copyright 2016, *Nat. Mater.*

Recently, Wang et al. [135] developed a diffusion type memristor composed of two electrodes and a SiN*_x_*O*_y_* film embedded with nano silver particles, wherein the film is placed between the two electrodes. The SiN*_x_*O*_y_* film is an insulator, but after electrification, under the action of heat and electric power, the positions of the silver particles arranged neatly on the film begin to scatter, gradually diffuse, and penetrate the film to finally form a cluster of conductive filaments. Current is passed from one electrode to the other. After turning off the power, the temperature drops and the nano silver particles are rearranged. The work of the memristor developed by the researchers is very similar to that of calcium ions in biological synapses, so the device can simulate the short-term plasticity of neurons, and the schematic diagram and circuit diagram of RRAM simulating synaptic memory are shown in Figure 5. In addition, the diffusion memristor can also be used as a selector with large transient nonlinearity, which has great research significance and application prospects.

### 4.3. Application of Optical Devices

By controlling the ratio of oxygen and nitrogen in the SiN*_x_*O*_y_* film, the refractive index and extinction coefficient of the SiN*_x_*O*_y_* film can be controlled, and it can be used as an optical waveguide material and an anti-reflection film.

Optical waveguide materials are generally required to have low transmission loss, single mode transmission, and the ability to fabricate a variety of active and passive devices on the same platform. The outstanding feature of planar optical waveguides based on SiN*_x_*O*_y_* and SiN is their ultra-low loss. Planar waveguides with attenuations less than 0.1 dB m^−1^ have been implemented, such as Baudzus et al. [136] studied phase shifters based on electro-optic (EO) polymers and SiN and SiN*_x_*O*_y_* waveguide materials systems, and found that SiN*_x_*O*_y_* and SiN can be combined with EO polymers and a fast adaptive phase shifter with very low attenuation can be made. The phase shifter has an attenuation of 0.8 dB cm^−1^ at 1550 nm and an EO efficiency factor of 27%, which can achieve lower loss and has important research significance. 

Optical waveguide materials, such as SiN*_x_*O*_y_* and SiN, also have applications in resonators. Compared with the straight waveguide structure, the ring resonator (RR) has the advantages of small footprint, high wavelength selectivity, and accumulated light intensity in the cavity, so that a large absorption is obtained in the case of a short absorption medium. Therefore, the microring resonator structure can achieve high responsiveness in the photodetector, achieving large extinction in the modulator. In recent years, the excellent performance of silicon waveguide photodetectors with integrated graphene has been experimentally verified. In a recent study of SiN microring resonators, Wang et al. [137] used a graphene-photonic integrated circuits (PICs) structure to fabricate microring resonators on the Si_3_N_4_ platform. Compared with the graphene-Si_3_N_4_ straight waveguide, the absorption of the ring resonator is increased, and the length of the required graphene is greatly shortened, and the quality factor of the prepared resonator is 0.282 × 10^5^–0.038 × 10^5^. In 2018, Jia et al. [138] developed a SiN*_x_*O*_y_*-based optical waveguide ring resonator (OWRR), which employed a liquid source CVD (LSCVD) method to deposit a SiN*_x_*O*_y_* film with a good refractive index. It is found that the SiN*_x_*O*_y_*-based resonator achieves a measured waveguide loss of 4.07 dB cm^−1^ and a quality factor of 0.93 × 10^5^ in the transverse electric (TE) mode, which provides a new idea for the preparation of other SiN*_x_*O*_y_*-based optical devices. The use of SiN and SiN*_x_*O*_y_* described above in ring resonators exhibits different ranges of quality factors, and in specific applications, is effectively selected based on the required bandwidth. In order to improve the controllability of the deposition process and the precise control of the SiN*_x_*O*_y_* film performance, PECVD and LPCVD methods are both used in the preparation process. When the refractive index is less than 1.7, it is suitable for deposition by PECVD. When the refractive index is greater than 1.7, the dependence of the refractive index of the SiN*_x_*O*_y_* film on the gas flow rate in PECVD deposition is large, which is not conducive to precise control. It must be deposited by LPCVD. The product rate is 1–10 nm/min, suitable for growing thin films with a thickness of 10–500 nm. The two deposition methods described above have thickness and refractive index uniformity and process repeatability of the resulting SiN*_x_*O*_y_* film deposited in the respective refractive index ranges, which can meet the requirements of high performance waveguides [139]. In addition to these, Trenti et al. [140] not only confirmed the low loss of SiN*_x_*O*_y_*, but also assumed that SiN*_x_*O*_y_* has thermal and nonlinear optical properties, and that SiN*_x_*O*_y_* is an excellent platform for nonlinear and quantum optical integrated photonic circuit design.

As an anti-reflective film, SiN*_x_*O*_y_* film can greatly reduce light reflection and loss, and increase efficiency when applied to photovoltaic devices such as solar cells and OLEDs. The excellent optical properties of SiN*_x_*O*_y_* film can increase the transmittance of light and reduce the reflectivity. Sapphire substrate is widely used in various photoelectric applications on glass substrate. However, sapphire has a low transmittance. In order to increase the transmittance of sapphire substrate and reduce the refractive index, Loka et al. [141] used RF magnetron sputtering to deposit a layer of SiN*_x_*O*_y_* film on the surface of sapphire, followed by annealing at a high temperature of 1099.85°C.The visible light transmittance is comparable with that of sodium-calcium glass. In this study, it is worth noting that after high temperature annealing, the nitrogen in the SiN*_x_*O*_y_* film disappears and the film is converted into SiO*_n_*. It is believed that the formation of SiO*_n_* and the high temperature annealing by increasing the oxygen content help to lower the refractive index and reflectivity. Moreover, SiN*_x_*O*_y_* film is used as an anti-reflection film on solar cells and OLEDs, which not only improves efficiency, but also protects the device due to its high resistance to corrosion and oxidation. However, in most preparation processes, composite films of SiN*_x_*O*_y_* and other components are usually double or even multilayered in order to obtain higher performance. In these studies, Nguyen et al. [142] developed a dual stack of SiN*_x_*O*_y_*/Al_2_O_3_ for n-type c-Si solar cells. They deposited a SiN*_x_*O*_y_* layer on the surface of Al_2_O_3_ as a coating by PECVD. It is found that the Al_2_O_3_/a-SiN*_x_*:H phase is excellent due to its excellent anti-reflection and front surface passivation of the Al_2_O_3_/SiON stack. The refractive index and high positive fixed charge of the SiN*_x_*O*_y_* cap layer in the Al_2_O_3_/SiN*_x_*O*_y_* stack are reduced, but the lifetime is significantly improved, and the optical properties are excellent, such as the average transmission in the entire wavelength range of 300–1100 nm. The rate is 93.8%, the average absorption rate is 0.33%, the energy conversion efficiency is increased from 17.55% to 18.34%, the short-circuit current density and open circuit voltage are also improved. Sahouane et al. [143] further investigated the application of multilayer anti-reflective coatings to reduce optical losses in solar cells. They deposited multilayer silicon nitride film and SiN*_x_*O*_y_* film by PECVD and found that the best reflectivity of six layers of film is 1.05%, and that of four layers is 3.26%. It is proved that multilayer deposition can greatly reduce reflectivity, reduce loss, and improve efficiency. Parashar et al. [144] found that the hybrid plasma structure formed by adding self-assembled silver-aluminum alloy nanoparticles to the SiN*_x_*O*_y_* film can also significantly reduce the reflectivity of silicon. The results showed that SiN*_x_*O*_y_*/Ag_2_Al nanoparticles reduce the average reflectance of silicon from 22.7% to 9.2%, and the SiN*_x_*O*_y_* cover layer reduces the reflectivity of silicon from 9.2% to 3.6% in the wavelength range of 300–1150 nm. By further enhancing photon management, the reflectivity decreased from 22.7% to ~3.6% in a 35 nm SiN*_x_*O*_y_*/Ag_2_Al nanoparticles /25 nm SiN*_x_*O*_y_* hybrid plasma structure, and when used as a battery, photocurrent and cell efficiency are improved to some extent. 

### 4.4. Application of Anti-Scratch Coating 

**Figure 6:**
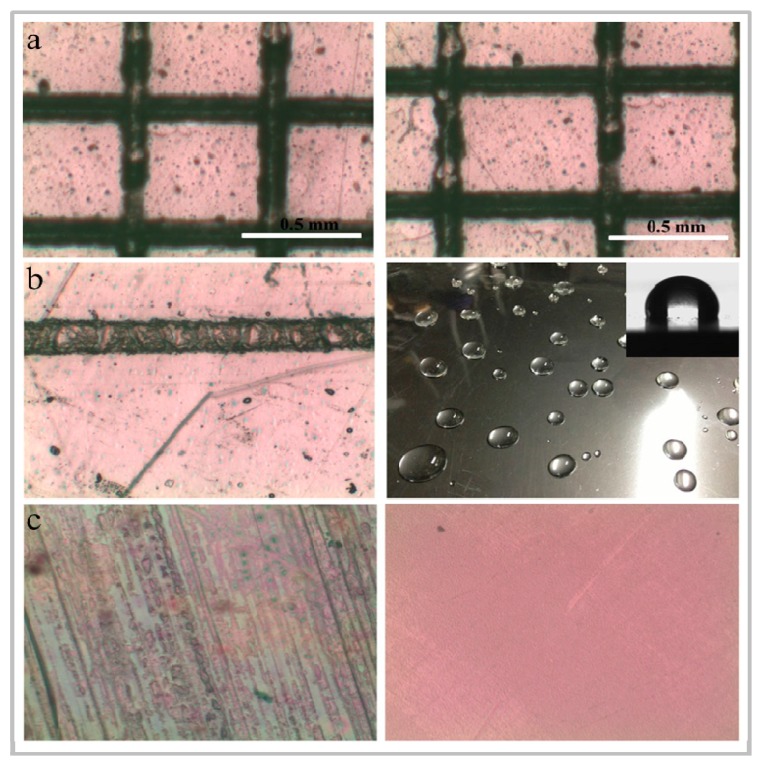
Scratch-resistant coating properties of the coated polyethylene terephthalate (PET) film. (**a**) Optical pictures of the coated films after cut-cross tape test (the left side shows a magnification of 1 time, and the right side shows a magnification of 10 times). (**b**) The left side shows the sample under the load of 1000 g left for the pencil scratch marks over 9 hours, and the water droplets on the coated PET film are shown in the right image. (**c**) Optical images of naked PET film. The PET film after coating is observed on the left and the right shows the coated PET film after wear test [149]. Copyright 2015, *Polym. Int*.

With the development of flexible optoelectronic devices, more and more transparent polymers are developed as flexible substrate in devices, and the polymer is widely used because of its advantages of light weight, low cost, high transparency, and easy to process and design [145,146]. However, for long-term use, the polymer is limited in many applications due to its poor scratch resistance, fragility, or influence on device. Researchers have further protected the device by depositing a coating on the surface of the transparent polymer to enhance its scratch resistance. In recent years, silica-based coatings (SiN*_x_*O*_y_* film) have been considered as promising transparent coatings for protection of transparent polymers. Lin et al. [147,148] deposited organo-silicon oxynitride (SiO*_x_*C*_y_*N*_z_*) film on a flexible polycarbonate (PC) substrate by low-temperature tetramethylsilane (TMS)-O_2_-N_2_ plasma polymerization at room temperature. They also studied the effect of N_2_ flow rate on scratch resistance of PC substrates, and found that the optimal flow rate of N_2_ is 3 sccm, and the scraping rate is 0% after scraping 200 times with steel wool under the pressure of 300 g. The SiO*_x_*C*_y_*N*_z_* film covered PC substrate has very good scratch resistance and provides a hard and smooth surface for flexible PC substrates. In addition, they also deposited SiO*_x_*C*_y_*N*_z_* film on reinforced carbon fiber reinforced polymer composites (FCFRPCs) by atmospheric pressure plasma low temperature polymerization to enhance the scratch resistance of the composites. There is a large amount of scratching (100%) on the original FCFRPC. By depositing SiO*_x_*C*_y_*N*_z_* film, the scratch resistance of FCFRPCs is significantly improved. After scratch testing, there are no scratches (0%), which is of great significance for the development of FCFRPCs. Furthermore, Zhang et al. [149] prepared a SiN*_x_*O*_y_* coating on glass and PET films by thermally annealing the inorganic polymer perhydropolysilazane (PHPS) between 60 and 200 °C. The results showed that the glass and PET film covered by SiN*_x_*O*_y_* film have higher hardness, stronger hydrophobicity and excellent adhesion. Additionally, the coated PET film exhibited high transparency and excellent scratch resistance in the visible wavelength range, and is advantageous as a hydrophobic scratch-resistant coating in optical devices, as shown in Figure 6.

The high hardness and high scratch resistance of the SiN*_x_*O*_y_* film make it widely used as a coating on transparent polymer and glass, which enhances the reliability of devices and has important research significance.

## 5. Conclusions

The SiN*_x_*O*_y_* film has important applications in optical devices, non-volatile memory, barrier materials, and scratch-resistant materials due to its good optoelectronic performance, mechanical strength, chemical stability, and barrier performance. The review focuses on the optical performance of SiN*_x_*O*_y_*, including luminescent performance and adjustable refractive index. This is a characteristic of SiN*_x_*O*_y_* film that is superior to conventional silicon-containing films such as Si_3_N_4_ and SiO_2_. Moreover, several methods for preparing SiN*_x_*O*_y_* film, including chemical deposition methods, sputtering methods, and nitrogen oxidation methods, which are mainly used at present, are reviewed and compared, and the advantages and disadvantages of different methods will help us design and select the preparation methods correctly.

As a new type of thin film, SiN*_x_*O*_y_* film has a wide range of research space in many aspects. We believe that with the maturity and development of various preparation methods, the application prospect of SiN*_x_*O*_y_* film will be brighter.

## Figures and Tables

**Table 1:** Comparison of materials, ratios, and deposition conditions in different chemical vapor deposition (CVD) methods.

Deposition Method	Precursor Gases	Ratio of Precursor Gases	Deposition Condition	Reference
PECVD	SiH_4_, N_2_O	SiH_4_/N_2_O = 0.05–0.125	200 °C, 97.09 Pa	[82]
RF-PECVD	SiH_4_, N_2_O, NH_3_	(NH_3_ + SiH_4_)/N_2_O = 0.64–3.22	120 Pa	[76]
ECR-PECVD	N_2_, O_2_, SiH_4_	O_2_/N_2_ = 0.03–0.1	-	[83]
IC-PECVD	N_2_, Ar, SiH_4_	N_2_/Ar = 0.0625–0.5	90–250 °C, 1–6 Pa	[84]
LPCVD	N_2_O, NH_3_ SiH_2_Cl_2_	N_2_O/NH_3_ = 4.8-	860 °C, 53.2 Pa	[85]

**Table 2:** Advantages and disadvantages of SiN*_x_*O*_y_* film prepared by PECVD, LPCVD, HTCVD and Photo-CVD methods.

Method	Advantages	Disadvantages
PECVD	Flexible operation method, High process repeatability, High step coverage, Low deposition temperature (<400 °C) [92]	High cost, High H content in film
LPCVD	Uniform film, Complete structure, Less pinhole defects, High deposition speed, Large-area preparation [80]	Low heating rate, Long reaction time, High deposition temperature (generally >550 °C)
HTCVD	Simple operation and operation, High reaction rate, Low H content in film and dense structure [80]	High deformation, Impaired interface performance
Photo-CVD	Low reaction temperature (≤250 °C), Smooth film surface, Less by-products [89]	High cost, Low film stability

**Table 3:** Preparation methods of SiN*_x_*O*_y_* film.

Method	Advantages	Disadvantages
CVD	High deposition rate Low deposition temperature Uniform film	Hydrogen content has an effect on electrical conductivity [111]
PVD	Low hydrogen content	Low deposition rate [112] Target poisoning is common
Oxynitridation	Relatively simple operation, large-scale preparation	Film thickness is difficult to control Toxicity of raw gas [110] Low N_2_ nitriding degree [113]
